# Vaccination Route as a Determinant of Protective Antibody Responses against Herpes Simplex Virus

**DOI:** 10.3390/vaccines8020277

**Published:** 2020-06-05

**Authors:** Clare Burn Aschner, Carl Pierce, David M. Knipe, Betsy C. Herold

**Affiliations:** 1 Department of Microbiology and Immunology, Albert Einstein College of Medicine, Bronx, NY 10461, USA; burn@mail.einstein.yu.edu (C.B.A.); Carl.Pierce@einsteinmed.org (C.P.); 2 Department of Microbiology, Blavatnik Institute, Harvard Medical School, Boston, MA 02115, USA; david_knipe@hms.harvard.edu; 3 Department of Pediatrics, Albert Einstein College of Medicine, Bronx, NY 10461, USA

**Keywords:** HSV vaccines, intradermal, intramuscular, ADCC

## Abstract

Herpes simplex viruses (HSV) are significant global health problems associated with mucosal and neurologic disease. Prior experimental vaccines primarily elicited neutralizing antibodies targeting glycoprotein D (gD), but those that advanced to clinical efficacy trials have failed. Preclinical studies with an HSV-2 strain deleted in gD (ΔgD-2) administered subcutaneously demonstrated that it elicited a high titer, weakly neutralizing antibodies that activated Fcγ receptors to mediate antibody-dependent cellular cytotoxicity (ADCC), and completely protected mice against lethal disease and latency following vaginal or skin challenge with HSV-1 or HSV-2. Vaccine efficacy, however, may be impacted by dose and route of immunization. Thus, the current studies were designed to compare immunogenicity and efficacy following different routes of vaccination with escalating doses of ΔgD-2. We compared ΔgD-2 with two other candidates: recombinant gD protein combined with aluminum hydroxide and monophosphoryl lipid A adjuvants and a replication-defective virus deleted in two proteins involved in viral replication, *dl*5-29. Compared to the subcutaneous route, intramuscular and/or intradermal immunization resulted in increased total HSV antibody responses for all three vaccines and boosted the ADCC, but not the neutralizing response to ΔgD and *dl*5-29. The adjuvanted gD protein vaccine provided only partial protection and failed to elicit ADCC independent of route of administration. In contrast, the increased ADCC following intramuscular or intradermal administration of ΔgD-2 or *dl*5-29 translated into significantly increased protection. The ΔgD-2 vaccine provided 100% protection at doses as low as 5 × 10^4^ pfu when administered intramuscularly or intradermally, but not subcutaneously. However, administration of a combination of low dose subcutaneous ΔgD-2 and adjuvanted gD protein resulted in greater protection than low dose ΔgD-2 alone indicating that gD neutralizing antibodies may contribute to protection. Taken together, these results demonstrate that ADCC provides a more predictive correlate of protection against HSV challenge in mice and support intramuscular or intradermal routes of vaccination.

## 1. Introduction

Herpes simplex virus 1 and 2 (HSV-1 and HSV-2) are prevalent human pathogens. HSV-1 infects approximately 67% of the population by 49 years of age and is the primary cause of oral and ocular disease, a leading cause of infectious corneal blindness and fatal infectious encephalitis and has emerged as the more common cause of genital disease in the developed world [1,2,3,4]. HSV-2 is estimated to affect over 400 million people worldwide, is the primary cause of genital disease in the developing world and a major risk factor for HIV acquisition and transmission [1]. 

The enormous global health burden of these two related viruses has resulted in extensive vaccine development efforts, which have primarily focused on the generation of neutralizing antibodies (nAbs) targeting the viral envelope glycoprotein D (gD) as the correlate of immune protection. One such vaccine was a recombinant gD-2 protein vaccine formulated with a proprietary aluminum hydroxide (alum) and monophosphoryl lipid A adjuvant, gD2−AS04 (GlaxoSmithKline, Brentford, UK). Despite promising preclinical studies and a Phase 3 clinical trial of serodiscordant couples demonstrating protection in HSV-1 and HSV-2 doubly seronegative women (but not men), a subsequent field trial found no protection against HSV-2 infection or disease in doubly-seronegative women [5,6]. The vaccine was administered intramuscularly at 0, 1 and 6 months. Another vaccine that has recently completed Phase I clinical trials is a replication-defective HSV-2 strain deleted in two genes involved in viral replication (*UL5* and *UL29*), designated *dl*5-29 (HSV529, Sanofi Pasteur, Lyon, France) [7]. In preclinical studies, the vaccine was safe, induced robust nAb and T cell responses and reduced the establishment of latency in the peripheral nerves [8,9,10,11,12,13]. The Phase I study also found that the vaccine was safe and elicited >4-fold increase in nAb responses in HSV seronegative participants, but no sustained increase in nAb responses in seropositive participants. Moreover, only a subset of participants elicited significant CD4 and even fewer CD8 T cell responses [7].

We have adopted a different strategy and evaluated a single-cycle virus deleted in gD; the primary target of the other vaccine candidates. In preclinical murine studies, this vaccine strain, ΔgD-2, elicited high-titer non-neutralizing Abs that activate Fc gamma receptors (FcγRs) to induce antibody-dependent cell-mediated cytotoxicity (ADCC). Two doses administered subcutaneously completely protected female and/or male mice against a lethal vaginal or skin challenge with clinical isolates of HSV-1 and HSV-2 and prevented the establishment of latency [14,15,16,17]. This protection was mediated by antibody effector function, as passive transfer protected wild type but not FcγR or Fc neonatal receptor (FcRn) knockout mice and T cell transfer failed to provide protection [14]. Moreover, vaccination of female mice protected their pups from subsequent HSV challenge in the first week of life [17]. In contrast to adjuvanted recombinant gD, ΔgD-2 boosted the total and the ADCC Ab responses in HSV-1 seropositive mice and prevented subsequent lethal HSV-2 superinfection [18]. 

Vaccine immunogenicity is impacted by how the viral antigens are presented (attenuated, replication-defective, single-cycle, inactivated virus or adjuvanted subunit protein), as well as the dose and route of administration. The immunization route is often based on pragmatic rather than immunologic considerations. Inactivated vaccines may be more immunogenic when delivered intradermally, for example, because of the abundance of immunostimulatory cells such as dendritic cells in the dermis [19]. Live viral vaccines are thought to be less influenced by the delivery route, although this has not been extensively tested. A prior study with *dl*5-29 found that two doses (10^6^ pfu/dose) delivered intramuscularly (im) provided greater immunogenicity and protection compared to subcutaneous (sc) or intradermal (id) vaccination [20,21]. Building on these observations, we extended our studies with ΔgD-2 and compared immunogenicity and efficacy using different routes and vaccine doses. We compared ΔgD-2, *dl*5-29 and recombinant gD protein combined with alum and MPL (gD-2-Alum/MPL), which is similar in composition to the gD2−AS04 vaccine, although in the clinical studies three rather than two doses were administered. We hypothesized that id administration would prove more immunogenic compared to sc for all three vaccines. 

## 2. Materials and Methods

### 2.1. Mice and Ethics Statement 

Age-matched (4–6 weeks old) female C57BL/6 (BL/6) mice were purchased from the Jackson Laboratory (JAX, Bar Harbor, ME, USA). The use of animals was approved by the Institutional Animal Care and Use Committee at the Albert Einstein College of Medicine, protocols 20180−504. 

### 2.2. Cell Lines, Viruses and Vaccines 

Vero (Green Monkey Kidney cells line, ATCC, Manassas, VA, USA), VD60 [22] and V5-29 [23] cells were grown in DMEM (Invitrogen, Carlsbad, CA, USA) supplemented with 10% FBS (Hyclone, Logan, UT, USA) and 1% penicillin-streptomycin (Invitrogen, Carlsbad, CA, USA). The clinical isolates used for viral challenges included HSV-2 (SD90) [24] and HSV-2 (4674). HSV-2 (4674) was obtained from the Montefiore Clinical Virology Lab [15,16]. The viral isolates were propagated and titered on Vero cells [15]. 

ΔgD-2 [14,15,16] was propagated in complementing VD60 cells, and titered both on VD60 and Vero cells. *Dl*5-29 [8] was propagated on complementing V5-29 cells [8]; and was also titered on complementing and non-complementing Vero cells. Recombinant gD-2 protein (5 µg) was provided by the Einstein Macromolecular Therapeutics Development Facility and adjuvanted with 150 µg alum (Imject Alum, Pierce Biotechnology, Rockland, IL, USA) and 12.5 µg MPL (Invivogen, San Diego, CA, USA) [16]. 

### 2.3. Vaccination and Challenge Protocol 

Female C57BL/6 mice were vaccinated subcutaneously, intramuscularly or intradermally on the right hind limb (two doses administered at three-week intervals) with 5 × 10^4^, 5 × 10^5^ or 5 × 10^6^ pfu ΔgD-2 or *dl*5-29 (based on viral titer on complementing cell line); 5 ug of rgD-2/Alum-MPL or a combination of 5 × 10^4^ pfu ΔgD2 and 5 µg of rgD-2/Alum-MPL. For intradermal vaccinations, a specialized intradermal microneedle designed for use in mice was used (Nanopass, Nes Ziona, ISR, Nes Ziona, Israel). Three weeks after the second vaccine dose, mice were challenged on the skin of the right flank with a 10 × lethal dose for 90% of animals (LD90) of HSV-2 SD90 [14]. Mice were monitored daily for epithelial and neurological disease and scored for skin and neurologic disease: (1) erythema at inoculation site; (2) spread to distant site, zosteriform lesions, edema; (3) ulceration, epidermal spread, limb paresis; (4) hind limb paralysis and (5) death. Mice were euthanized at a score of 4 and assigned a score of 5 the following day.

### 2.4. ELISA for HSV-Specific Antibodies

Total or isotype-specific HSV-binding IgG was measured by ELISA using serum collected one week following the second dose of vaccine. ELISA plates were coated with lysates of Vero cells infected with HSV-2 (G) at a multiplicity of infection (MOI) of 0.1 for 24 h or uninfected Vero cell lysates as control. Serial dilutions of serum in duplicate were incubated with coated plates overnight at 4 °C, and bound IgG was quantified using biotin-labeled secondary Abs (BD Pharmingen, CA, USA). Background binding to uninfected Vero cell lysates was subtracted from binding to HSV-infected Vero cell lysates to quantify HSV-specific binding.

### 2.5. FcγR Activation Assay

FcγRIV activation was determined using the murine FcγRIV ADCC Reporter Bioassay (Promega, Madison, WI, USA) [15,16]. Target Vero cells were infected with HSV-2 (SD90) at an MOI of 0.1 for 12 h. Infected or uninfected control cells were transferred to white, flat-bottomed 96-well plates and incubated with heat-inactivated serum from vaccinated or control immunized mice (1:5 dilution in DMEM) for 15 min at room temperature. Murine FcγRIV-expressing effector cells were added for 6 h at 37 °C 5% CO_2_ and FcγRIV activation was detected by the addition of luciferin substrate. Plates were read in a SpectraMax M5^e^ (Molecular Devices, San Jose, CA, USA). Fold induction was calculated relative to luciferase activity in the absence of serum.

### 2.6. Neutralization Assay

Neutralizing titers were determined by plaque reduction assay [14,15,16]. Serial 2-fold dilutions of heat-inactivated serum (in duplicate) were incubated with the virus (50 pfu/well) for 1 h at 37 °C and then applied to Vero cell monolayers for 1 h at 37 °C. Cells were fixed with methanol and stained with Giemsa after a 48h incubation. Plaques were counted and the neutralization titer was defined as the highest dilution to result in a 50% reduction in plaque numbers.

### 2.7. Quantification of Viral DNA in Neuronal Tissue by Quantitative PCR 

At the time of euthanasia (when mice succumbed to disease or day 14 post-HSV-2 challenge); sacral nerve tissue was extracted and DNA was isolated using the Qiagen Blood and Tissue DNA isolation kit (Qiagen, Hilden, Germany). 10 ng of DNA per sample was loaded, and primers and probes specific for HSV-2 gB were used to quantify HSV DNA (HSV-2 forward primer sequence 5′-TGCAGTTTACGTATAACCACATACAGC-3′; HSV-2 reverse primer sequence 5′-AGCTTGCGGGCCTCGTT-3′ and HSV-2 probe sequence 5′-CGCCCCAGCATGTCGTTCACGT-3′) [25]. Mouse β actin was used as a loading control (Applied Biosystems, Foster City, CA, USA), and qPCR was run in an Applied Biosystems QuantStudio 7 Flex (Thermo Fisher, Foster City, CA, USA). Based on a standard curve, this assay consistently detected copy numbers greater than or equal to 4. Samples with fewer than 4 copies detected were considered negative [14,15,16]. 

### 2.8. Cell Isolation and Flow Cytometry

Peripheral blood was collected by retro-orbital bleed, pipetted into 5 mL prewarmed ACK lysing buffer (Lonza BioWhittaker, Walkersville, MD, USA) and incubated for 7 min at 37 °C. Following lysis, cells were washed 2× in PBS without calcium and magnesium. For splenocyte isolation, spleens were isolated from vaccinated animals and mechanically digested by pressing through a 70 µm cell strainer. Cells were pelleted by centrifugation and resuspended in 2 mL ACK lysing buffer. After 7 min at 37 °C, RPMI was added and cells were pelleted by centrifugation. Cells were subsequently washed and resuspended in RPMI for further processing.

For ex-vivo stimulation, 2 × 10^6^ splenocytes per 200 µL of RPMI + 10% FBS were plated in a U-bottom 96-well plate. Cells were treated with PHA (5 µg/mL) or 1 × 10^6^ PFU UV-inactivated HSV-2 SD90 and incubated at 37 °C for 18 h. Brefeldin A (BioLegend, San Diego, CA, USA) was added for the final 5 h of stimulation. For UV inactivation of virus, HSV-2 SD90 was diluted in RPMI in a 24-well dish and exposed to a hand-held UV light positioned 4 inches above the plate for 30 min. Cells were then processed for extracellular and intracellular staining for flow cytometry. 

For flow cytometry analysis, 1–2 × 10^6^ cells per 100 µL were incubated with Zombie Near-IR fixable viability dye and TruStain FcX (anti-mouse CD16/CD32) antibody for 10 min at room temperature. For surface staining, cells were stained with anti-CD90.2-BV510, CD4-BV785, CD8-BV711, CD11a-APC, CD49d-APC/Fire750, KLRG1-BV605 and CD62L-BV570 (all BioLegend, San Diego, CA, USA) in a mixture of FACS buffer and brilliant stain buffer (BD Biosciences, Franklin Lakes, NJ, USA) for 30 min at room temperature (RT) per the manufacturer’s instructions. Cells were then washed and fixed by incubating in 200 µL 2% PFA for 20 min at room temperature, and subsequently permeabilized by incubating for 7 min in 0.3% Triton X-100. For intracellular staining, cells were incubated in 100 µL of a cocktail of anti-IFN-γ-PE, TNF-BV570 and IL2−-PerCP/Cy5.5 (BioLegend, San Diego, CA, USA) for 30 min at 4 °C. Following staining, cells were washed and passed through a 40 µm cell strainer, prior to analysis on a 5-laser Cytek Aurora flow cytometer. Per sample, 50,000 live CD90.2^+^ cells were collected, and data analysis was carried out using FlowJo (BD Biosciences, Franklin Lakes, NJ, USA). Proportions of cytokine producing cells were determined by Boolean gating.

### 2.9. Statistical Analysis 

Analyses were performed using GraphPad Prism version 8.2.1 software (GraphPad Software Inc., San Diego, CA, USA). A *p* value of 0.05 was considered statistically significant. Survival curves were compared using the Gehan–Breslow–Wilcoxon test; other results were compared using ANOVA or mixed effects analyses as indicated. 

## 3. Results

### 3.1. Dose and Delivery Route Influence HSV Vaccine Immunogenicity

**Figure 1:**
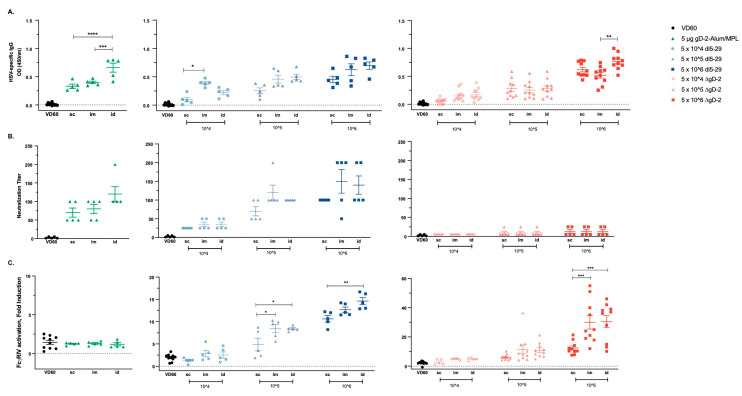
Immunogenicity of viral and adjuvanted subunit herpes simplex virus (HSV) vaccines is modulated by the vaccination route. C57BL/6 mice were vaccinated twice, three weeks apart with 5 × 10^4^, 5 × 10^5^ or 5 × 10^6^ pfu/mouse of *dl*5-29 or ΔgD-2, or 5 µg gD-2-Alum/MPL subcutaneously (sc), intramuscularly (im) or intradermally (id). One week following the second immunization, mice were retro-orbitally bled and serum was tested for (**A**) total HSV-specific IgG by ELISA, (**B**) neutralization titer and (**C**) FcγRIV activation by the NFAT-luciferase reporter assay. Note the difference in the y-axis scale in (C). *n* = 5 mice per group for gD-2-Alum/MPL and *dl*5-29 in a single experiment; *n* = 5 mice per group, two independent experiments for ΔgD-2. Asterisks denote significance, * *p* < 0.05, ** *p* < 0.01, *** *p* < 0.001, **** *p* < 0.0001 by ANOVA.

To determine whether the dose and/or route of delivery impacted immunogenicity, mice were prime-boost immunized with increasing doses of ΔgD-2 or *dl*5-29 (5 × 10^4^, 5 × 10^5^ or 5 × 10^6^ pfu/dose based on titer on complementing cell lines) or with 5 µg of gD protein adjuvanted with alum and MPL via the sc, im or id route. The total HSV-specific (ELISA), neutralizing and ADCC response (measured using murine FcγRIV activation as a surrogate) were quantified in serum obtained one-week post-boost. The adjuvanted gD protein vaccine elicited a significantly higher total HSV ELISA antibody response when delivered id compared to im or sc (*p* < 0.001). The total HSV-specific Ab response to *dl*5-29 and ΔgD-2 increased with escalation of the dose, but there were few differences comparing the route of administration at each dose; the im route induced a significantly higher response compared to sc for *dl*5-29 at a dose of 5 × 10^4^ pfu/mouse (*p* < 0.05) and the id route induced a higher Ab response compared to im for ΔgD-2 at a dose of 5 × 10^6^ pfu/mouse (*p* < 0.01, ANOVA; Figure 1A).

Consistent with the increase in total HSV-specific Abs, there was a nonsignificant increase in the neutralizing titer following id administration of rgD-/Alum-MPL (Figure 1B). The neutralizing response to *dl*5-29 increased with dose, but not when comparing the route of administration. ΔgD-2, as anticipated from prior studies, induced little or no neutralizing Ab response regardless of dose or route of administration. In contrast, ΔgD-2 elicited the most potent ADCC response compared to the other vaccines, which increased with dose and was significantly greater at the 5 × 10^6^ dose when comparing id or im to sc administration. The adjuvanted gD protein vaccine induced no ADCC response relative to control serum regardless of route of administration. The *dl*5-29 vaccine induced an intermediate ADCC response, which was the highest following id administration of 5 × 10^6^ pfu (median 15-fold) compared to the 30-fold FcγRIV activation elicited by 5 × 10^6^ pfu of ΔgD2− administered im or id (Figure 1C). 

### 3.2. Differences in Immunogenicity Translate to Differences in Protection Following Lethal Skin Challenge

**Figure 2:**
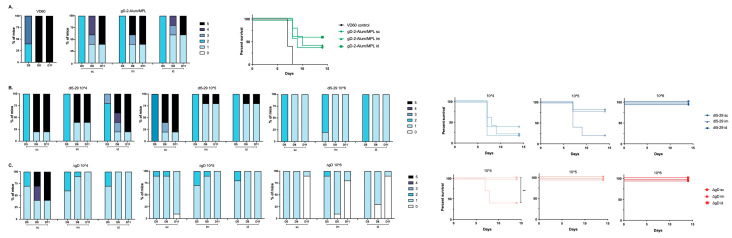
Differences in immunogenicity based on vaccine dose and route translate to differences in protection. Female C57BL/6 mice were vaccinated twice, three weeks apart with 5 × 10^4^, 5 × 10^5^ or 5 × 10^6^ pfu/mouse of *dl*5-29 or ΔgD-2 or 5 µg gD-2-Alum/MPL subcutaneously (sc), intramuscularly (im) or intradermally (id). Three weeks following the second vaccination, mice were challenged on the skin with 10 × LD90 HSV-2 (SD90). Disease scores over time (left panels) are shown for gD-2-Alum/MPL (**A**), *dl*5-29 (**B**) and ΔgD-2 (**C**). Percentage survival is shown in right panels. *n* = 5 mice per group for gD2−Alum/MPL and *dl*5-29 in a single experiment; *n* = 5 mice per group, two independent experiments for ΔgD2−. For survival curves, ** *p* < 0.01 by the Gehan–Breslow–Wilcoxon test.

The prime-boost vaccinated mice were challenged on the skin with a 10 × LD90 dose of the clinical isolate of HSV-2, SD90, which has been previously shown to be consistently lethal in murine models [24]. Mice were monitored for two weeks for signs of disease and were euthanized if signs of severe skin or neurologic disease were observed as previously described (Figure 2) [14,15,16]. There was a modest but not significant increase in protection afforded by the adjuvanted protein vaccine when administered id compared to im or sc (Figure 2A), which parallels the increased ELISA and nAb responses (Figure 1). The route of administration had no significant impact on survival following 5 × 10^4^ dose of *dl*5-29, which was not protective, but both the im and id routes provided greater protection than the sc route following a 5 × 10^5^ dose of *dl*5-29 (80% versus 20%), which paralleled the significant increase in ADCC. All three routes were fully protective at the highest vaccine dose (Figure 2B). The only breakthrough in survival with ΔgD-2 was observed with a dose of 5 × 10^4^ administered sc. Complete protection against lethality was observed at all other doses and routes (Figure 2C). When examining the association between ADCC and survival across the total population independent of dose, route or vaccine (*n* = 145), 93/96 mice with a 4.5-fold increase in mFcγRIV activation survived compared to 14/49 with <4.5-fold increase (*p* < 0.0001, chi-square). 

**Figure 3:**
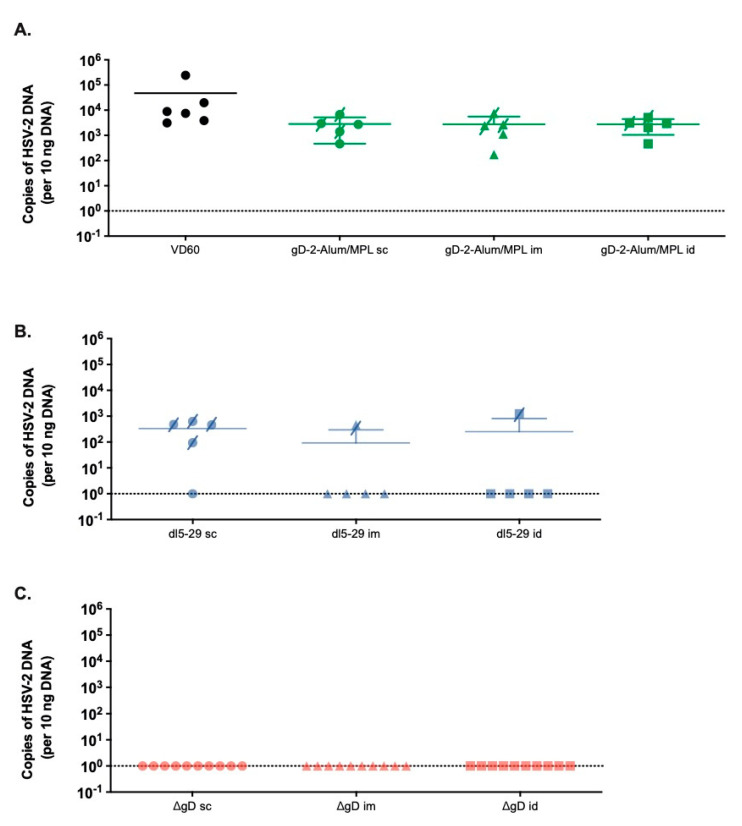
HSV DNA detection in the sacral nerve parallels survival data. Female C57BL/6 mice vaccinated with 5 µg rgD-2-Alum/MPL, or 5 × 10^5^ pfu/mouse of *dl*5-29 or ΔgD-2 by the sc, im or id routes were challenged in the skin with 10 × LD90 HSV-2 (SD90). Following the challenge, mice were monitored daily for fourteen days and sacral nerve tissue was harvested at the time of death for mice that succumbed to challenge, or at D14 post challenge for surviving animals. HSV DNA in the sacral ganglia was assessed by qPCR and the number of copies of HSV-2 DNA per 10 ng of DNA is shown in (**A**) for rgD-2-Alum/MPL, (**B**) *dl*5-29 and (**C**) ΔgD-2. Mice that succumbed to challenge are indicated by a crossed through symbol. There were no significant differences based on the vaccine route (ANOVA).

To assess whether the route of vaccination impacted the ability of vaccines to prevent the establishment of latency, HSV viral DNA was quantified in ganglia at the time of death or on day 14 post-challenge. Despite the increase in the Ab response following id vaccination with adjuvanted gD protein, there was no reduction in viral DNA recovered from ganglia following any route of immunization. The results with *dl*5-29 and ΔgD-2 at the 5 × 10^5^ dose paralleled the disease scores and survival data. Only 1/5 of mice immunized im or id compared to 4/5 of mice immunized sc with *dl*5-29 had HSV DNA detected in the ganglia. No viral DNA was recovered in mice vaccinated by any route with the same dose of ΔgD-2 (Figure 3). 

### 3.3. ΔgD-2 Vaccination Induces Robust CD4 and CD8 T Cell Memory Responses

**Figure 4:**
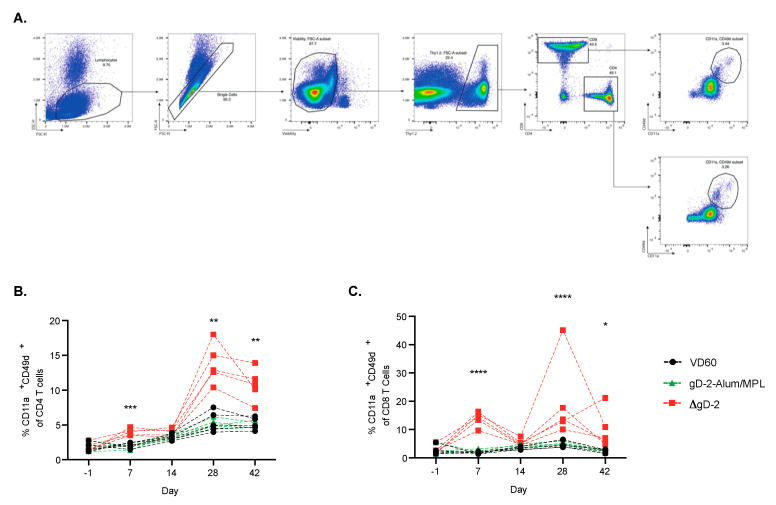
Kinetics of the T cell response following HSV vaccination. Female C57BL/6 mice were vaccinated im twice, three weeks apart, with 5 × 10^5^ pfu/mouse of ΔgD-2 or 5 µg gD-2−Alum/MPL. Before vaccination (day 1) and at week 1 and 2 following prime and boost, mice were retro-orbitally bled and assessed for CD11a^+^ CD49d^+^ activatedCD4 and CD8 T cells. (**A**) The gating strategy is shown for the assessment of (**B**) CD4 and (**C**) CD8 T cell activation. Data was analyzed by mixed effects analysis, * *p* < 0.5, ** *p* < 0.01, *** *p* < 0.001, **** *p* < 0.0001; *n* = 5 mice per group.

**Figure 5:**
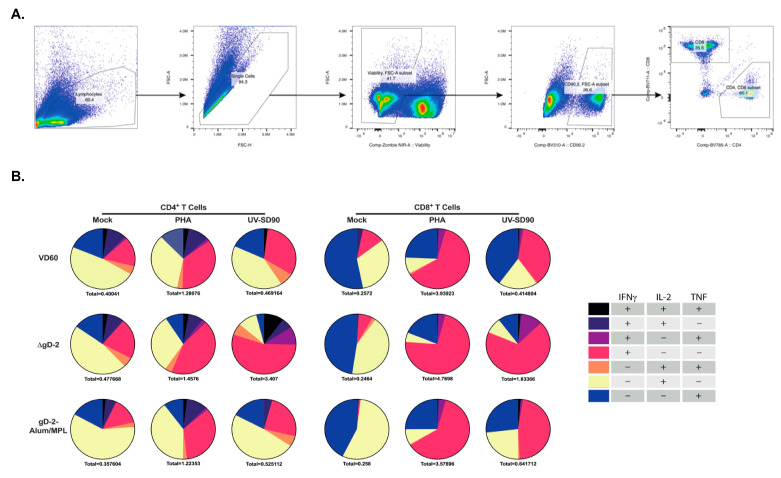
ΔgD-2 vaccination induces polyfunctional CD4 and CD8 T cells that produce IFN-γ, TNF and IL-2 in response to HSV-2 stimulation. Female C57BL/6 mice were vaccinated im twice, three weeks apart, with 5 × 10^5^ pfu/mouse of ΔgD-2 and 5 µg gD-2-Alum/MPL. Splenocytes from vaccinated mice were collected two weeks following boost vaccination and stimulated with PHA or UV-inactivated HSV-2 (SD90) for 18 h with Brefeldin A treatment before staining and flow cytometric analysis for the production of IFN-γ, TNF and IL-2. The gating strategy is shown in (**A**), and cytokine responses for CD4 and CD8 T cells in (**B**). Pie charts represent the population producing at least one cytokine. Total cytokine-producing cells as a percentage of live CD4 and CD8 T cells is indicated below each graph; *n* = 5 mice per group.

To further phenotype the difference in immune response to ΔgD-2 and rgD-2-Alum/MPL, which trigger functionally distinct Abs, mice were prime-boost vaccinated im with 5 × 10^5^ pfu/mouse of ΔgD-2 or 5 µg gD-2−Alum/MPL at three week intervals. T cell responses were assessed in the peripheral blood prior to vaccination (day 1) and at the indicated times post prime and boost. ΔgD-2 induced activated CD4 and CD8 T cells following both prime and boost vaccination as measured by quantifying CD11a+CD49+ CD4 and CD8 T cells. In contrast, there was little detectable T cell response to the adjuvanted protein vaccine (Figure 4A–C). The splenocytes from these mice were harvested on day 42 and stimulated with UV-inactivated SD90 or phytohemagglutinin (PHA) as a viability control to assess the cytokine responses (Figure 5A). Significantly more IFN-γ, TNF and IL-2 producing CD4+ T cells were observed when splenocytes isolated from ΔgD-2, but not rgD-2-Alum/MPL vaccinated mice were stimulated with inactivated virus compared to unstimulated cells (Figure 5B). The response was greater than observed with PHA. There was also a non-significant increase in cytokine-producing CD8 T cell responses to the ΔgD2− vaccine compared to unstimulated cells (Figure 5B).

### 3.4. Combination of Low Dose ΔgD-2 with rgD-2 Provides Additive Protection

**Figure 6:**
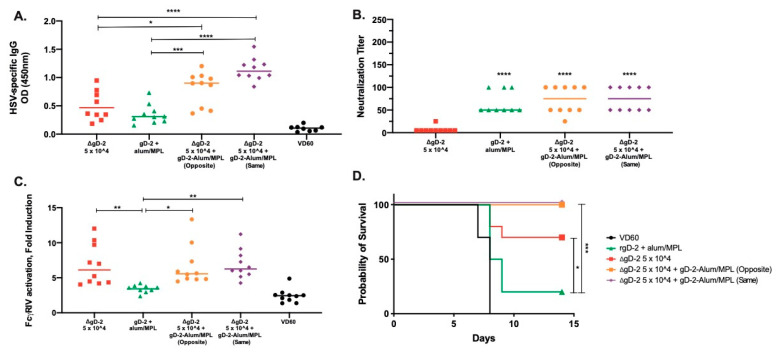
The generation of neutralizing antibody enhances protection by low-dose ΔgD-2. Female C57BL/6 mice were subcutaneously vaccinated twice, three weeks apart with 5 × 10^4^ pfu/mouse of ΔgD-2, 5 µg gD-2-Alum/MPL or a combination of both vaccines delivered on opposite flanks (opposite) or at the same site (same). One week after the second vaccination, mice were retro-orbitally bled and serum was assessed for (**A**) total HSV-specific IgG by ELISA, (**B**) neutralizing titer and (**C**) FcγRIV activation. (**D**) Three weeks after the second vaccination, mice were challenged on the skin with a 10 × LD90 dose of HSV-2 (SD90). Percentage survival is shown. *n* = 5 mice per group, two independent experiments. (A–C) * *p* < 0.05, ** *p* < 0.01, *** *p* < 0.001, **** *p* < 0.0001 by ANOVA. For survival curves, * *p* < 0.05, *** *p* < 0.001 by the Gehan–Breslow–Wilcoxon test.

To determine whether the combination of ΔgD-2 and rgD-2-Alum/MPL is beneficial or antagonistic, mice were vaccinated sc with a dose of ΔgD-2 that is not fully protective (5 × 10^4^ pfu/mouse), 5 μg of gD-2-Alum/MPL or a combination of both vaccines delivered on opposite or the same flank. We used the less efficient route of vaccination to accentuate any potential beneficial effects. Both combinations significantly increased the total HSV-specific antibody response compared to either vaccine alone (Figure 6A). The combinations had no additive or antagonistic effect on the nAb response to rgD2−/Alum-MPL (Figure 6B) or the ADCC response to ΔgD-2 (Figure 6C) and resulted in 100% protection against a 10 × LD90 skin challenge with HSV-2 (SD90), compared to the 20% and 60% protection observed with administration of rgD-2-Alum/MPL or ΔgD-2, respectively (Figure 6D). There was no difference when the combination was administered on the opposite or the same flank.

## 4. Discussion

Clinical studies with HSV vaccine candidates have proven disappointing, despite promising preclinical data with vaccines designed to elicit neutralizing antibody responses primarily targeting gD. Preclinical studies with ΔgD-2 have challenged the reliance on neutralizing Abs and have demonstrated that subcutaneous vaccination with 10^5^ (or higher) pfu of ΔgD-2 reproducibly provides complete protection against lethal skin, vaginal or ocular challenge with clinical isolates of HSV-1 or HSV-2 [14,15,16,26]. Protection is mediated by ADCC rather than neutralizing Abs, as evidenced by passive transfer studies. Immune serum from ΔgD-2, but not rgD-2-Alum/MPL vaccinated mice completely protects naïve wild-type, but not FcγR (common, FcγRIV or neonatal receptor) knockout mice from lethal challenge [14,15,16,17,18].

The current studies, which were conducted in female mice challenged on the skin rather than intravaginally because a skin challenge does not require pretreatment with medroxyprogesterone and is reflective of the clinical presentation on genital skin [27,28], provide further evidence that ADCC provides a more predictive correlate of immune protection compared to neutralizing responses in mice. The increase in protection observed by increasing the dose and route of delivery of *dl*5-29 was associated with a significant increase in the ADCC, but not the neutralizing response. Moreover, the only dose and route of vaccination with ΔgD-2 that did not provide 100% protection against a 10 × LD90 challenge with SD90, sc immunization with 10^4^ pfu, elicited a mean ADCC response of 2.8-fold (FcγRIV) activation. Only 3 out of 96 mice with an FcγRIV fold increase >4.5 succumbed to the high dose lethal challenge regardless of vaccine dose or delivery route. 

Both the im and id routes of vaccination induced significantly higher total and/or ADCC responses compared to the sc route. The observation that im and id are more immunogenic than sc is consistent with studies with other vaccines, but a link between route of administration and Ab function (ADCC versus neutralizing) has not been previously described. Improved immunogenicity via the im or id routes could reflect longer antigen retention, differential exposure to antigen presenting cells resident in the dermis and/or greater access to lymphatic drainage [29,30,31,32,33]. For example, intramuscular administration of trivalent inactivated influenza vaccine resulted in higher antibody responses than subcutaneous vaccination in elderly subjects [34]. However, similar Ab. and T cell responses were reported with live attenuated measles, mumps and rubella vaccination delivered sc or im [35]. Despite technical difficulties delivering consistent doses via the intradermal route, intradermal rabies vaccination has been a standard following a World Health Organization recommendation in 1992 because a lower dose achieves comparable immunogenicity [36]. Intradermal vaccination is presumed to activate a stronger dendritic cell-mediated response thus requiring a lower antigenic dose [37]. However, we observed no significant difference in ADCC responses or vaccine protection comparing id or im immunization routes at any of the doses for *dl*5-29 or ΔgD-2, suggesting that the id route does not provide a dose advantage for these vaccines. We did, however, observe a statistically significant increase in total HSV-specific Ab responses to the subunit vaccine with the id route of administration, which resulted in a nonsignificant increase in neutralizing titer and in vaccine protection.

In addition to dose and delivery route, the vaccine composition also influences immunogenicity, as evidenced by the exclusive neutralizing response to the gD subunit vaccine, non-neutralizing, FcγR-mediated response to ΔgD-2, and a combination of both neutralizing and non-neutralizing responses elicited by *dl*5-29. The absence of any neutralizing Ab following ΔgD-2 immunization likely reflects the absence of the dominant target of nAbs in mice. In other studies, we found that depletion of the gD-specific Ab from *dl*5-29 immune serum resulted in a significant reduction in neutralizing, but not ADCC titers, indicating that gD is not a target of the ADCC response (Burn Aschner and Herold, manuscript under review). IgG subclass switching to IgG2, which has the strongest affinity for mFcγRIV and is associated with ADCC in mice, requires interactions within the germinal center between antigen presenting cells, T cells and B cells [38]. Consistent with the requirement for T cells in the generation of potent ADCC responses, we documented robust activation of CD4 and CD8 T cells after prime and boost vaccination, while gD-2-alum/MPL elicited little T cell activation. CD8 responses to prime and boost with either vaccine contracted more rapidly than CD4 T cells. The precise mechanisms for these differences requires further study, but they are consistent with kinetics of CD4 and CD8 responses to other unrelated viral vaccines [39,40,41]. Further studies are required to allow for a more detailed analysis of CD4 and CD8 T cell activation, including stimulation with HSV-infected antigen presenting cells. Stimulation of the memory T cells harvested from the ΔgD-2, but not rgD-2-Alum/MPL vaccinated mice, with inactivated virus result in IFN-γ, TNF and IL-2 production, which was particularly robust for CD4+ T cells. These differences support the notion that vigorous T cell responses contribute to the generation of ADCC responses.

A combination of adjuvanted rgD-2 and a low dose of ΔgD-2 delivered simultaneously at the same or opposite flank did not interfere with the immunogenicity of either vaccine and was more protective than rgD2−Alum/MPL alone. This is consistent with our superinfection murine studies, which showed that pre-existing gD neutralizing Abs did not interfere with the immunogenicity of ΔgD-2. Vaccination of HSV-1 seropositive mice with ΔgD-2 boosted the ADCC (but not the neutralizing) Ab response and resulted in complete protection if the mice were subsequently challenge with a lethal dose of HSV-2 [18]. Thus, while nAbs to gD alone are not sufficient to protect mice (or to date, humans), a combination of both types of responses could be beneficial. One reason for the incomplete protection mediated by nAbs may be the ability of HSV to evade neutralization by spreading directly from cell-to-cell [42]. However, it is important to note that delivering the recombinant gD protein at the same time as ΔgD-2 is different from having gD present in the viral envelope. In other studies, we found that envelope gD interferes with the generation of IgG2 subclass switched Abs through interactions with herpesvirus entry mediator (HVEM), also known as tumor necrosis factor receptor superfamily member 14 (Burn Aschner and Herold, manuscript under review). This likely contributes to the lower levels of ADCC generated by *dl*5-29 as shown in the current study as well as the low levels generated in response to sublethal infection [17,18].

## 5. Conclusions

Taken together, the current studies provide further evidence that ADCC is an important correlate of immune protection. Although we initially hypothesized that the intradermal route of delivery would prove more immunogenic for all three vaccines, this was only observed with the gD protein subunit vaccine. Both im and id routes provided similar antibody responses and protection with ΔgD-2 and *dl*5-29. Overall, ΔgD-2 induced the highest ADCC responses and the most potent protection against a lethal challenge and latency. It remains to be determined how well these findings in the murine models translate to the clinic.

## Data and Materials Availability

All data supporting the findings of this study are available within the manuscript. Materials available on request.

## References

[1] Looker K.J., Magaret A.S., Turner K.M.E., Vickerman P., Gottlieb S.L., Newman L.M. (2015). Global Estimates of Prevalent and Incident Herpes Simplex Virus Type 2 Infections in 2012. PLoS ONE.

[2] Lafferty W.E., Downey L., Celum C., Wald A. (2000). Herpes simplex virus type 1 as a cause of genital herpes: Impact on surveillance and prevention. J. Infect. Dis..

[3] Roberts C.M., Pfister J.R., Spear S.J. (2003). Increasing Proportion of Herpes Simplex Virus Type 1 as a Cause of Genital Herpes Infection in College Students. Sex. Transm. Dis..

[4] Xu F., Sternberg M.R., Kottiri B.J., McQuillan G.M., Lee F.K., Nahmias A.J., Berman S.M., Markowitz L.E. (2006). Trends in herpes simplex virus type 1 and type 2 seroprevalence in the United States. JAMA.

[5] Stanberry L.R., Spruance S.L., Cunningham A.L., Bernstein D.I., Mindel A., Sacks S., Tyring S., Aoki F.Y., Slaoui M., Denis M. (2002). GlaxoSmithKline Herpes Vaccine Efficacy Study Group Glycoprotein-D-adjuvant vaccine to prevent genital herpes. N. Engl. J. Med..

[6] Belshe R.B., Leone P.A., Bernstein D.I., Wald A., Levin M.J., Stapleton J.T., Gorfinkel I., Morrow R.L.A., Ewell M.G., Stokes-Riner A. (2012). Herpevac Trial for Women Efficacy results of a trial of a herpes simplex vaccine. N. Engl. J. Med..

[7] Dropulic L.K., Oestreich M.C., Pietz H.L., Laing K.J., Hunsberger S., Lumbard K., Garabedian D., Turk S.P., Chen A., Hornung R.L. (2019). A Randomized, Double-Blinded, Placebo-Controlled, Phase 1 Study of a Replication-Defective Herpes Simplex Virus (HSV) Type 2 Vaccine, HSV529, in Adults with or without HSV Infection. J. Infect. Dis..

[8] Da Costa X.J.E.A., Kramer M.F., Zhu J., Brockman M.A., Knipe D.M. (2000). Construction, phenotypic analysis, and immunogenicity of a UL5/UL29 double deletion mutant of herpes simplex virus 2. J. Virol..

[9] Da Costa X.J.E.A., Morrison L.A., Knipe D.M. (2001). Comparison of Different Forms of Herpes Simplex Replication-Defective Mutant Viruses as Vaccines in a Mouse Model of HSV-2 Genital Infection. Virology.

[10] Hoshino Y., Pesnicak L., Dowdell K.C., Lacayo J., Dudek T., Knipe D.M., Straus S.E., Cohen J.I. (2008). Comparison of immunogenicity and protective efficacy of genital herpes vaccine candidates herpes simplex virus 2 dl5-29 and dl5-29-41L in mice and guinea pigs. Vaccine.

[11] Hoshino Y., Dalai S.K., Wang K., Pesnicak L., Lau T.Y., Knipe D.M., Cohen J.I., Straus S.E. (2004). Comparative Efficacy and Immunogenicity of Replication-Defective, Recombinant Glycoprotein, and DNA Vaccines for Herpes Simplex Virus 2 Infections in Mice and Guinea Pigs. J. Virol..

[12] Hoshino Y., Pesnicak L., Dowdell K.C., Burbelo P.D., Knipe D.M., Straus S.E., Cohen J.I. (2009). Protection from herpes simplex virus (HSV)-2 infection with replication-defective HSV-2 or glycoprotein D2 vaccines in HSV-1-seropositive and HSV-1-seronegative guinea pigs. J. Infect. Dis..

[13] Bernard M.-C., Barban V., Pradezynski F., de Montfort A., Ryall R., Caillet C., Londoño-Hayes P. (2015). Immunogenicity, Protective Efficacy, and Non-Replicative Status of the HSV-2 Vaccine Candidate HSV529 in Mice and Guinea Pigs. PLoS ONE.

[14] Petro C., González P.A., Cheshenko N., Jandl T. (2015). Herpes simplex type 2 virus deleted in glycoprotein D protects against vaginal, skin and neural disease. eLife.

[15] Petro C.D., Weinrick B., Khajoueinejad N., Burn C., Sellers R., Jacobs W.R., Herold B.C. (2016). HSV-2 ΔgD elicits FcγR-effector antibodies that protect against clinical isolates. JCI Insight.

[16] Burn C., Ramsey N., Garforth S.J., Almo S., Jacobs W.R., Herold B.C. (2018). An HSV-2 single-cycle candidate vaccine deleted in glycoprotein D, ΔgD-2, protects male mice from lethal skin challenge with clinical isolates of HSV-1 and HSV-2. J. Infect. Dis..

[17] Kao C.M., Goymer J., Loh L.N., Mahant A., Burn Aschner C., Herold B.C. (2020). Murine Model of Maternal Immunization Demonstrates Protective Role for Antibodies That Mediate Antibody-Dependent Cellular Cytotoxicity in Protecting Neonates from Herpes Simplex Virus Type 1 and Type 2. J. Infect. Dis..

[18] Burn Aschner C., Knipe D.M., Herold B.C. (2020). Model of vaccine efficacy against HSV-2 superinfection of HSV-1 seropositive mice demonstrates protection by antibodies mediating cellular cytotoxicity. NPJ Vaccines.

[19] Romani N., Flacher V., Tripp C.H., Sparber F., Ebner S., Stoitzner P. (2012). Targeting skin dendritic cells to improve intradermal vaccination. Curr. Top. Microbiol. Immunol..

[20] Diaz F., Gregory S., Nakashima H., Viapiano M.S., Knipe D.M. (2018). Intramuscular delivery of replication-defective herpes simplex virus gives antigen expression in muscle syncytia and improved protection against pathogenic HSV-2 strains. Virology.

[21] Delagrave S., Hernandez H., Zhou C., Hamberger J.F., Mundle S.T., Catalan J., Baloglu S., Anderson S.F., DiNapoli J.M., Londoño-Hayes P. (2012). Immunogenicity and Efficacy of Intramuscular Replication-Defective and Subunit Vaccines against Herpes Simplex Virus Type 2 in the Mouse Genital Model. PLoS ONE.

[22] Ligas M.W., Johnson D.C. (1988). A herpes simplex virus mutant in which glycoprotein D sequences are replaced by beta-galactosidase sequences binds to but is unable to penetrate into cells. J. Virol..

[23] Da Costa X.J.E.A., Jones C.A., Knipe D.M. (1999). Immunization against genital herpes with a vaccine virus that has defects in productive and latent infection. Proc. Natl. Acad. Sci. USA.

[24] Dudek T.E., Torres-Lopez E., Crumpacker C., Knipe D.M. (2011). Evidence for Differences in Immunologic and Pathogenesis Properties of Herpes Simplex Virus 2 Strains from the United States and South Africa. J. Infect. Dis..

[25] Namvar L., Olofsson S., Bergström T., Lindh M. (2005). Detection and typing of Herpes Simplex virus (HSV) in mucocutaneous samples by TaqMan PCR targeting a gB segment homologous for HSV types 1 and 2. J. Clin. Microbiol..

[26] Ramsey N.L.M., Visciano M., Hunte R., Loh L.N., Burn Aschner C., Jacobs W.R., Herold B.C. (2020). A single-cycle glycoprotein D deletion viral vaccine candidate, ΔgD-2, elicits polyfunctional antibodies that protect against ocular herpes simplex virus. J. Virol..

[27] Gupta R., Warren T., Wald A. (2007). Genital herpes. Lancet.

[28] Tanton C., Weiss H.A., LeGoff J., Changalucha J., Clayton T.C., Ross D.A., Belec L., Hayes R.J., Watson-Jones D. (2011). Patterns of herpes simplex virus shedding over 1 month and the impact of acyclovir and HIV in HSV-2-seropositive women in Tanzania. Sex. Transm. Infect..

[29] Wahl M., Hermodsson S. (1987). Intradermal, subcutaneous or intramuscular administration of hepatitis B vaccine: Side effects and antibody response. Scand. J. Infect. Dis..

[30] Bryan J.P., Sjogren M.H., Perine P.L., Legters L.J. (1992). Low-dose intradermal and intramuscular vaccination against hepatitis B. Clin. Infect. Dis..

[31] Rahman F., Dahmen A., Herzog-Hauff S., Böcher W.O., Galle P.R., Löhr H.F. (2000). Cellular and humoral immune responses induced by intradermal or intramuscular vaccination with the major hepatitis B surface antigen. Hepatology.

[32] Belshe R.B., Newman F.K., Cannon J., Duane C., Treanor J., Van Hoecke C., Howe B.J., Dubin G. (2004). Serum antibody responses after intradermal vaccination against influenza. N. Engl. J. Med..

[33] Van Damme P., Oosterhuis-Kafeja F., Van der Wielen M., Almagor Y., Sharon O., Levin Y. (2009). Safety and efficacy of a novel microneedle device for dose sparing intradermal influenza vaccination in healthy adults. Vaccine.

[34] Cook I.F., Barr I., Hartel G., Pond D., Hampson A.W. (2006). Reactogenicity and immunogenicity of an inactivated influenza vaccine administered by intramuscular or subcutaneous injection in elderly adults. Vaccine.

[35] Gillet Y., Habermehl P., Thomas S., Eymin C., Fiquet A. (2009). Immunogenicity and safety of concomitant administration of a measles, mumps and rubella vaccine (M-M-RvaxPro) and a varicella vaccine (VARIVAX) by intramuscular or subcutaneous routes at separate injection sites: A randomised clinical trial. BMC Med..

[36] Laurent P.E., Bourhy H., Fantino M., Alchas P., Mikszta J.A. (2010). Safety and efficacy of novel dermal and epidermal microneedle delivery systems for rabies vaccination in healthy adults. Vaccine.

[37] Dubois B., Bridon J.M., Fayette J., Barthélémy C., Banchereau J., Caux C., Brière F. (1999). Dendritic cells directly modulate B cell growth and differentiation. J. Leukoc. Biol..

[38] Peng S.L., Szabo S.J., Glimcher L.H. (2002). T-bet regulates IgG class switching and pathogenic autoantibody production. Proc. Natl. Acad. Sci. USA.

[39] Rai D., Pham N.-L.L., Harty J.T., Badovinac V.P. (2009). Tracking the total CD8 T cell response to infection reveals substantial discordance in magnitude and kinetics between inbred and outbred hosts. J. Immunol..

[40] McDermott D.S., Varga S.M. (2011). Quantifying antigen-specific CD4 T cells during a viral infection: CD4 T cell responses are larger than we think. J. Immunol..

[41] Bovay A., Nassiri S., Maby-El Hajjami H., Marcos Mondéjar P., Akondy R.S., Ahmed R., Lawson B., Speiser D.E., Fuertes Marraco S.A. (2020). Minimal immune response to booster vaccination against Yellow Fever associated with pre-existing antibodies. Vaccine.

[42] Sattentau Q. (2008). Avoiding the void: Cell-to-cell spread of human viruses. Nat. Rev. Microbiol..

